# Trophic Interactions and Distribution of Some Squaliforme Sharks, Including New Diet Descriptions for *Deania calcea* and *Squalus acanthias*

**DOI:** 10.1371/journal.pone.0059938

**Published:** 2013-03-25

**Authors:** Matthew R. Dunn, Darren W. Stevens, Jeffrey S. Forman, Amelia Connell

**Affiliations:** 1 School of Biological Sciences, Victoria University of Wellington, Wellington, New Zealand; 2 National Institute of Water and Atmospheric Research Limited, Greta Point, Wellington, New Zealand; 3 York Bay, Lower Hutt, New Zealand; Aristotle University of Thessaloniki, Greece

## Abstract

Squaliforme sharks are a common but relatively vulnerable bycatch in many deep water fisheries. Eleven species of squaliforme shark are commonly caught at depths of 200–1200 m on Chatham Rise, New Zealand, and their diversity suggests they might occupy different niches. The diets of 133 *Deania calcea* and 295 *Squalus acanthias* were determined from examination of stomach contents. The diet of *D. calcea* was characterised by mesopelagic fishes, and *S. acanthias* by benthic to pelagic fishes, but was more adaptive and included likely scavenging. Multivariate analyses found the most important predictors of diet variability in *S. acanthias* were year, bottom temperature, longitude, and fish weight. The diet of the nine other commonly caught squaliforme sharks was reviewed, and the spatial and depth distribution of all species on Chatham Rise described from research bottom trawl survey catches. The eleven species had a variety of different diets, and depth and location preferences, consistent with niche separation to reduce interspecific competition. Four trophic groups were identified, characterised by: mesopelagic fishes and invertebrates (*Centroselachus crepidater*, *D. calcea*, and *Etmopterus lucifer*); mesopelagic and benthopelagic fishes and invertebrates (*Centroscymnus owstoni*, *Etmopterus baxteri*); demersal and benthic fishes (*Centrophorus squamosus*, *Dalatias licha*, *Proscymnodon plunketi*); and a generalist diet of fishes and invertebrates (*S. acanthias*). The trophic levels of the species in each of the four groups were estimated as 4.18–4.24, 4.20–4.23, 4.24–4.48, and 3.84 respectively. The diet of *Oxynotus bruniensis* and *Squalus griffini* are unknown. The different niches occupied by different species are likely to influence their vulnerability to bottom trawl fisheries. Some species may benefit from fisheries through an increased availability of scavenged prey.

## Introduction

Deep-sea sharks are abundant and widely distributed on Chatham Rise, New Zealand [1], where they are a common bycatch in longline and trawl fisheries [2]. Sharks share a number of biological characteristics that make them susceptible to over-utilisation [3], but the status of populations on Chatham Rise is unknown [2], [4]. Of the squaliforme shark species commonly caught by deep water (>400 m) research bottom trawls on Chatham Rise, *Proscymnodon plunketi* and *Dalatias licha* are listed by the IUCN as “near threatened”, and *Squalus acanthias* and *Centrophorus squamosus* are listed as “vulnerable”; the other seven commonly caught species, *Centroscymnus owstoni*, *Centroselachus crepidater*, *Deania calcea*, *Etmopterus baxteri*, *Etmopterus lucifer*, *Oxynotus bruniensis* and *Squalus griffini*, are listed as “least concern” or “data deficient”. Although there is international concern over the vulnerability of sharks to commercial exploitation [5]–[8], research bottom trawl surveys on Chatham Rise suggest shark population sizes have not declined substantially over the last 20 years [9].

Squaliforme sharks are expected to be important predators on the continental slope, yet the diet of most species is poorly known. The diversity of sympatric shark species on Chatham Rise suggests they might occupy different niches. Some sharks are known to predate directly upon species targeted by important commercial fisheries on Chatham Rise, for example on hoki *Macruronus novaezelandiae* eggs [10] or juveniles and adults [11], and others compete with commercial finfishes for food resources [11]. Better understanding of the trophic role of sharks can therefore be potentially valuable to fishery managers. For example, assessments of stock status and sustainable yields may be improved by estimating variability in natural mortality rate (*M*), where *M* is estimated from predator (i.e., including sharks) abundance and diet [12], [13]. As top predators, sharks demonstrating adaptive foraging may also help to bring stability to ecosystems impacted by fishing [14]. Understanding the trophic role of sharks may also help identify threats to their own species conservation [15].

The shovelnose dogfish, *Deania calcea*, and the spiny dogfish, *Squalus acanthias*, are the most commonly caught shark species during research bottom trawl surveys on Chatham Rise, with maximum catch rates of about 1.5 t km^−2^ and 5.4 t km^−2^ respectively [9]. In New Zealand waters, reported annual commercial catches in recent years have been about 300 t of *D. calcea*, and 3000–7000 t of *S. acanthias* [4], but true catches are likely to be higher as many sharks are discarded and not recorded [2]. Some aspects of the biology of both species are known from international studies, and *S. acanthias* is a relatively well studied, but the diet of neither species on Chatham Rise has been described before.

**Figure 1:**
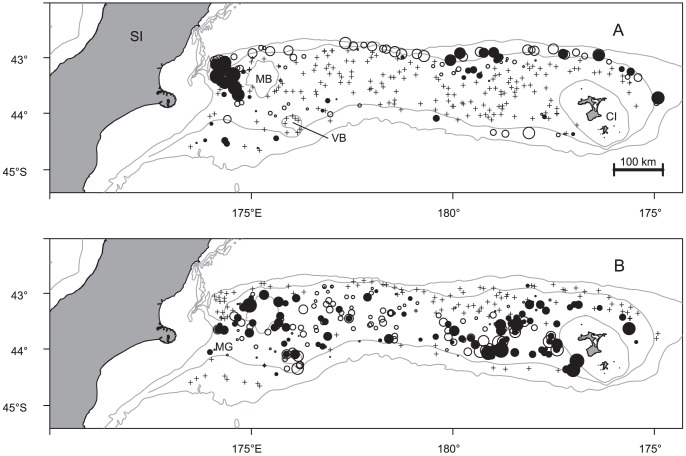
Locations on Chatham Rise of the research trawl tows (crosses), and tows where a) *Deania calcea*, and b) *Squalus acanthias* were caught (open circles) and non-empty stomach samples were obtained (filled circles). Circle sizes are proportional to catch (max. 189 kg for *D. calcea* and 264 kg for *S. acanthias*) and stomach sample size (max. 51 for *D. calcea* and 49 for *S. acanthias*). Grey lines show the 200 m, 600 m, and 1000 m isobaths; SI, South Island of New Zealand; CI, Chatham Islands; MG, Mernoo Gap; MB, Mernoo Bank; VB, Veryan Bank.

Chatham Rise is an undersea ridge which runs eastwards for about 1000 km from the east coast of the South Island of New Zealand (Fig. 1). The subtropical front (STF) forms over Chatham Rise, where the mixing of subantarctic and subtropical water masses produces a region of heightened primary productivity [16], supporting abundant mesopelagic biomass [17]. Pronounced ecosystem changes across the STF on Chatham Rise have been correlated with changes in diet for several fish species [18]–[20], presumably because of variations in environmental conditions and prey availability.

This paper first provides a quantitative description of the diets of *D. calcea* and *S. acanthias* on Chatham Rise. We then review and classify the trophic role of *D. calcea*, *S. acanthias* and nine other squaliforme sharks commonly caught on Chatham Rise, using other New Zealand and international studies. We combine our trophic classification with an analysis of each species' spatial and depth distribution on Chatham Rise, allowing us to evaluate multiple components of potential niche separation for all eleven species. We discuss the veracity of the proposed niche separation, and how this may affect each species' interaction with commercially targeted fish stocks and fisheries.

## Materials and Methods

### Ethics

This study was exempt from ethical approval by the NIWA Animal Ethics Committee.

### Diet descriptions for *Deania calcea* and *Squalus acanthias*

Samples were obtained from stratified-random research bottom trawl surveys on the Chatham Rise during December 2004–January 2005 and December 2005–January 2006 [9]. The sampling strata were defined by location and depth, covering about 146 855 km^2^, depths between 200 and 1200 m, and all but the far southeast corner of Chatham Rise. A full–wing bottom trawl was towed by *RV Tangaroa* at each station for c. 3 nautical miles (5.6 km), at a speed of 3.5 knots (6.5 km/h), and at about 100 stations per year.

The sharks were sampled opportunistically, from most tows where they were caught. Fish were measured (total length (TL) to the nearest mm), weighed (to the nearest 5 g), and sexed. Fish with obviously regurgitated or everted stomachs were not sampled. At sea, stomachs were sealed by fixing a cable-tie around the oesophagus, then the oesophagus was cut in front of the tie, the intestines cut below the pyloric sphincter, and the stomach removed, labelled, frozen at −20°C and returned to the laboratory.

Each stomach was thawed, the wet weight of stomach and contents recorded, the stomach contents removed and rinsed with water, and the wet weight of the empty stomach recorded. Recognisable prey items were then identified. For each prey category, the individual prey items were counted, and the wet–weight recorded after removal of surface water by blotting paper.

The contribution of different prey items to the diet was determined by numerical importance (%N), frequency of occurrence (%F), mass (%W) and percentage index of relative importance (%IRI) [21], [22]. Bootstrap methods, consisting of 1000 replicates of random samples, with replacement, of stomachs from the original data set, stratified by tow, were used to estimate 95% confidence intervals around the dietary statistics [23].

To conduct analyses of diet variability the prey items were aggregated into taxonomic categories. The prey categories were chosen to achieve maximum prey resolution, whilst maintaining sample size, and varied with the ability to identify different prey taxa. Invertebrate prey were classified to phylum, class, or order level, except for shrimps and prawns which were classified together as Natantia, and Astacidea, Achelata, Anomura, and Brachyura which were classified together as Reptant Decapoda; vertebrate prey were classified to order or family level, except for discarded fish offal which was classified together as ‘discarded fish’. Discarded fish offal was recognisable as cleanly severed fish heads and/or tails, or filleted fish frames. The unidentifiable prey, sand, rocks, shell fragments, nematode and trematode parasites found in the stomachs, and prey classified as well digested, were excluded from detailed analyses. The knowledge of prey ecology was generally poor, and considered insufficient to allow a convincing functional grouping of prey.

To assess the adequacy of the samples, the cumulative diversity (Brillouin index of diversity, *H*) of categorised stomach contents was plotted against the cumulative number of stomachs containing food [24]. The mean and 95% credible interval were calculated from 1000 curves based upon different random orders of the stomachs. The sample was considered adequate for analyses of diet variability if the mean sample diversity (*H*) was ≥95% of the asymptotic diversity (*H_A_*), estimated from a fitted curve of the form *H* = *a*n/(1+*b*n) [25].

Distance-based linear model (DistLM) analysis in PRIMER v6 [26] was used to identify which of the potential predictors explained most of the variability in diet. Data were first standardised, then square-root transformed, and a dissimilarity matrix calculated using Bray-Curtis distances. The potential predictors were fish total length, weight, and sex, and the tow year, time of day, depth, latitude and longitude (the latter three all a mean of the start and finish positions), and bottom water temperature. Significant and relevant correlations between predictors are reported in the results. The most significant predictors were selected using the “best” selection method, which used both the Akaike Information Criterion (AIC) and Bayesian Information Criterion (BIC) [27], with the most parsimonious model selected by plotting the top 50 models chosen using each criterion as a scatter plot, and selecting the models which appeared in both criteria and had the lowest combined criterion scores [26]. The results of the DistLM analysis were a marginal test, fitting each predictor individually, and a conditional test, fitting each predictor conditional on the predictor(s) already in the model. To further investigate the effects of the predictors identified from the DistLM analysis, the continuous predictors were binned, with bin limits chosen so that the number of observations in each bin was approximately equal. The target number of samples in each bin was sufficiently large to describe >95% of the estimated diversity of the overall diet. The binned data were averaged (mean of normalised proportions of prey species), square-root transformed, and then the characteristic prey groups identified with SIMPER (similarity percentages) [28]. The actual mean percentage weight of the prey groups identified by SIMPER was then calculated to show the main differences in diet composition between bins.

### Review of squaliforme shark diet

The diet of common squaliforme sharks on Chatham Rise was reviewed from previously published New Zealand and worldwide studies. The species reviewed were *Centroscymnus owstoni*, *Centrophorus squamosus*, *Centroselachus crepidater*, *Dalatias licha*, *Deania calcea*, *Etmopterus baxteri*, *Etmopterus lucifer*, *Oxynotus bruniensis*, *Proscymnodon plunketi*, *Squalus acanthias*, and *Squalus griffini*. There were insufficient data for all of these species on Chatham Rise to complete quantitative diet comparisons. Three species that were rarely encountered were not included in the diet review; *Centroscymnus coelolepis* (2 occurrences in 21 years of Chatham Rise research trawl surveys, [9]), *Etmopterus pusillus* (1 occurrence), and *Etmopterus molleri* (1 occurrence). For each species and study where quantitative diet composition data were provided, the trophic level was estimated following Cortés [22]. Where unidentified or unidentifiable prey were included as part of the diet composition statistics these categories were ignored, and the identified prey re-scaled. Trophic level was not estimated where prey descriptions were incomplete, for example where ‘minor’ prey were not listed. The trophic level was estimated from prey occurrence descriptions only when this could be interpreted as percentage numerical importance (%N). The overall tropic level for each species was a weighted (by sample size) mean of the individual studies [22]. The review by Cortés [22] was not included in the present review as this would duplicate some data sources.

### Distribution analyses

The spatial distribution of the shark species included in the diet review was then evaluated by estimating species abundance by location (latitude, longitude, and depth) on Chatham Rise. Standardised tow-by-tow catch rates (kg km^−2^) were obtained from 21 annual stratified-random research bottom trawl surveys [9]. For each species, the mean catch rate per 0.3° latitude and longitude cell was plotted, as well as the catch rate against depth. Although the average catch rate would better represent by the median than the mean (because catch rates are usually approximately log-normally distributed), the mean was preferred as it ensured cells with a single non-zero catch had an average that was greater than zero. The trend in catch rate at depth was evaluated by fitting LOESS regressions. LOESS smooths the data by fitting a local quadratic regression using weighted least squares, and was implemented using the *loess* function in R (http://www.R-project.org).

## Results

### Diet of *Deania calcea*

**Figure 2:**
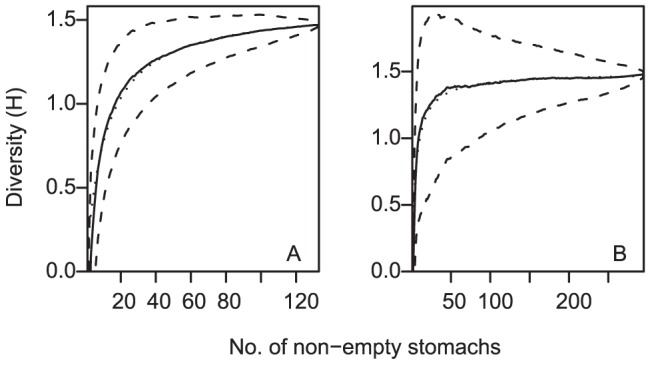
Cumulative diversity of prey categories (solid lines) and 95% credible intervals (broken lines) in the analyses of diet of a) *Deania calcea*, and b) *Squalus acanthias*. Dotted lines are the fitted curves from which asymptotic diversities were estimated.

*Deania calcea* were not caught at the majority of tow locations, and were sampled predominantly from two areas; the western end of Chatham Rise, and on the north Chatham Rise east of 180° longitude (Fig. 1a). Of 314 specimens examined, 100 (32%) had empty stomachs. The number of prey items per stomach ranged between 1 and 7, with 55% containing only a single prey item. Prey remains were all unidentifiable or well digested in 81 stomachs, leaving 133 for detailed analyses of diet. The 133 specimens were sampled from a median depth of 573 m (range 420–727 m), and had a median length of 81.9 cm TL (range 37.7–109.8 cm TL), and a TL (cm) to weight (g) relationship of W = 0.00078×TL^3.34^ (R^2^ = 0.99; Std. errors 0.00017 and 0.049 respectively) The diversity of prey categories reached 90% of the estimated asymptote after 94 stomachs, but did not reach 95% of the asymptote (Fig. 2a). As a result, although the sample described diet reasonably well, it was not considered large enough for analyses of diet variability.

The diet of *D. calcea* was characterised by teleost fishes, the majority of which could not be identified, and natant decapods (Table S1). Myctophids were the most frequent and numerous of the identifiable fish prey, with at least five species consumed. However in terms of prey weight myctophids were relatively unimportant, because they were relatively small, and other prey categories of larger fish were more important, particularly merlucciids and macrourids, as well as various squids.

### Diet of *Squalus acanthias*

*Squalus acanthias* were caught in most tows within the depth range 200–400 m, and were sampled predominantly from two areas; the western end of Chatham Rise, and to the west of the Chatham Islands (Fig. 1b). Of 550 specimens examined, 63 (11%) had empty stomachs. The number of prey items per stomach ranged between 1 and 252, with 61% containing only a single prey item. Prey remains were all unidentifiable or well digested in 192 stomachs, leaving 295 for detailed analyses of diet. The 295 specimens were sampled from a median depth of 380 m (range 209–761 m), and had a median length of 74.4 cm TL (range 55.2–105.7 cm TL), and a TL (cm) to weight (g) relationship of W = 0.0032×TL^3.08^ (R^2^ = 0.98; Std. errors 0.00049 and 0.035 respectively) The diversity of prey categories reached 95% of the estimated asymptote after 47 stomachs (Fig. 2b), indicating the sample was large enough for analyses of diet variability.

The diet of *S. acanthias* was characterised by teleost fishes, and although the majority of the fish prey could not be identified, at least 24 species were consumed, having habits ranging from benthic to mesopelagic (Table S2). Other important prey were salps, euphausiids, and squids. The most important fish prey category was scavenged jack mackerel (*Trachurus* spp.), followed by Merluccidae (entirely hoki *Macruronus novaezelandiae*) and macrourids (at least four species). Salps and euphausiids were the most numerous prey, but squids were most important by prey weight, followed by unidentified fish, scavenged fish, hoki, and octopods. The squid prey were most frequently identified as arrow squid *Nototodarus* spp., and the scavenged fish were heads and/or tails only of jack mackerel *Trachurus* spp. or hoki.

The DistLM analysis indicated significant relationships between diet and several of the predictors (Table 1), with the sequential model having the predictors year, bottom temperature, longitude, and fish weight, together explaining 13.2% of the deviance.

**Table 1:** *Squalus acanthias* results of the DistLM analysis marginal and conditional tests, using all stomachs containing prey (*n* = 295).

Variable	d.f.	*P*	r^2^
**Marginal model**			
Length	2	0.001	0.032
Weight	2	0.001	0.036
Sex	2	0.006	0.010
Year	3	0.001	0.057
Time of day	2	0.080	0.006
Depth	2	0.001	0.026
Latitude	2	0.001	0.020
Longitude	2	0.001	0.025
Bottom temperature	2	0.001	0.030
**Conditional (sequential) model**			
Year	3	0.001	0.057
+ Bottom temperature	5	0.001	0.089
+ Longitude	6	0.001	0.111
+ Weight	7	0.001	0.132

The diet of *S. acanthias* was characterised by salps, copepods, euphausiids and squids in 2005 (Table 2). In 2006 the diet featured more squids and reptant decapods (which were predominantly *Metanephrops challengeri*), and in 2007 the diet featured more discarded fishes and macrourids. There were no strong correlations between year and any other predictor (r^2^≤0.23).

**Table 2:** *Squalus acanthias* diet by year.

	2005	2006	2007
*n*	145	33	117
Salpidae	24.0^c^	3.6	12.1^b^
Copepoda	14.4^b^	1.5	0.0
Euphausiacea	15.5^b^	1.5	10.2^a^
Reptant Decapoda	5.5	17.6^a^	2.6
Macrouridae	3.3	0.1	12.3^b^
Discarded fishes	1.4	9.1	25.8^c^
Teuthoidea	9.9^a^	47.6^c^	10.3^a^

Mean of standardised percent prey weight within each year, for the prey types together contributing at least 90% of the SIMPER within group similarity for one or more groups. SIMPER percentage contribution to within group similarity:

a 3–10%;

b 10–30%;

c >30%; no superscript, not identified by SIMPER as characteristic for that group; *n*, sample size.

On the western flank of Chatham Rise (173.9–177.4°E), the diet of *S. acanthias* was characterised by euphausiids and squids, and to a lesser extent by salps, macrourids, and reptant decapods (Table 3). Moving eastwards across Chatham Rise, the diet featured more fishes, including merlucciids and macrourids, and then more copepods, salps, and discarded fishes. Squids were least important towards the centre of Chatham Rise (177.8°E – 177.8°W). Longitude was moderately correlated with latitude (r^2^ = 0.39), which was caused by the absence of samples from the southeast Chatham Rise, but weakly correlated with other predictors (r^2^≤0.21).

**Table 3:** *Squalus acanthias* diet by longitude.

	173.9–175.0°E	175.3–177.4°E	177.8°E–178.8°W	178.9–177.8°W	177.9–175.6°W
*n*	54	57	62	63	59
Salpidae	9.6^a^	3.8	26.9^c^	19.3^b^	23.8^c^
Copepoda	0.0	0.0	12.4^b^	21.5^c^	0.2
Euphausiacea	24.6^c^	24.3^c^	2.8	3.4	6.4
Reptant Decapoda	11.3^a^	4.3	4.5	3.2	5.8
Merlucciidae	4.0	7.0^a^	3.6	4.8	0.0
Macrouridae	8.1^a^	13.0^b^	8.1^a^	1.6	2.4
Discarded fishes	3.7	8.7^a^	6.8^a^	23.8^c^	15.3^b^
Teuthoidea	18.7^b^	14.0^b^	9.3^a^	7.9	22.4^c^

Mean of standardised percent prey weight within each longitude group, for the prey types together contributing at least 90% of the SIMPER within group similarity for one or more groups. SIMPER percentage contribution to within group similarity:

a 3–10%;

b 10–30%;

c >30%; no superscript, not identified by SIMPER as characteristic for that group; *n*, sample size.

The diet of small (≤1090 g) *S. acanthias* was characterised by salps, small crustaceans, and myctophids (Table 4). Euphausiids featured in the diet of all sizes of *S. acanthias*. As *S. acanthias* got larger, the diet featured less salps, the crustacean component changed from amphipods and copepods to reptant decapods, the fish component changed from myctophids to macrourids, discarded fishes, and eventually to merlucciids, and the cephalopod component featured squids throughout, but octopods only in larger (≥1425 g) *S. acanthias*. Fish weight was moderately correlated with latitude (r^2^ = 0.42; larger fish on south Chatham Rise) and sex (r^2^ = 0.40), but weakly correlated with other predictors (r^2^≤0.19).

**Table 4:** *Squalus acanthias* diet by fish weight.

	655–1090	1095–1415	1425–2395	2400–3030	3100–5000
*n*	60	58	58	59	60
Salpidae	31.5^c^	26.9^c^	17.6^c^	5.1	4.2
Amphipoda	6.2	6.8^a^	3.9	2.1	0.5
Copepoda	11.6^a^	15.6^b^	0.6	3.4	5.0
Euphausiacea	19.0^b^	11.3^a^	8.5^a^	11.6^b^	8.6^a^
Reptant Decapoda	3.1	3.9	8.1^a^	5.1^a^	8.2
Merlucciidae	0.0	0.0	4.9	3.4	11.0^a^
Myctophidae	8.3^a^	0.1	4.1	0.0	1.7
Macrouridae	0.5	5.2	8.8^a^	13.5^b^	4.6
Discarded fishes	0.0	8.6	8.6^a^	22.0^c^	20.4^b^
Teuthoidea	6.9	13.8^b^	14.8^b^	8.5^a^	27.2^c^
Octopoda	0.0	0.0	5.7^a^	4.9	3.6

Mean of standardised percent prey weight within each fish weight (g) group, for the prey types together contributing at least 90% of the SIMPER within group similarity for one or more groups. SIMPER percentage contribution to within group similarity:

a 3–10%;

b 10–30%;

c >30%; no superscript, not identified by SIMPER as characteristic for that group; *n*, sample size.

In cooler water (≤7.5°C), the diet was characterised by salps, amphipods, macrourids, and squids (Table 5). The diet then changed with increasing water temperature, and included copepods at 7.6–8.1°C, at ≥8.2°C featured more discarded fishes and squids, and at ≥10.0°C was dominated by euphausiids. Bottom temperature was strongly correlated with depth (r^2^ = 0.85; cooler in deeper water) and moderately correlated with latitude (r^2^ = 0.50; cooler to the south), and weakly correlated with other predictors (r2≤0.20).

**Table 5:** *Squalus acanthias* diet by bottom temperature.

	6.3–7.5	7.6–8.1	8.2–9.1	9.2–9.9	10.0–11.3
*n*	58	66	58	65	48
Salpidae	30.1^c^	23.5^c^	13.1^b^	10.7^a^	5.5
Amphipoda	11.0^a^	4.7	0.3	0.1	3.7
Copepoda	2.9	27.3^c^	2.6	0.0	0.2
Euphausiacea	3.7	4.3	15.3^b^	1.6	41.7^c^
Macrouridae	12.8^b^	1.9	5.2	6.6^a^	6.5
Discarded fishes	6.9	10.6^a^	14.2^b^	21.5^c^	4.1
Teuthoidea	11.5^a^	5.4	20.9^c^	23.3^c^	9.6^a^

Mean of standardised percent prey weight within each bottom temperature (°C) group, for the prey types together contributing at least 90% of the SIMPER within group similarity for one or more groups. SIMPER percentage contribution to within group similarity:

a 3–10%;

b 10–30%;

c >30%; no superscript, not identified by SIMPER as characteristic for that group; *n*, sample size.

### Review of squaliforme shark diet

The sample sizes used to describe diet were generally small (*n*<50) (Table 6). There was a paucity of diet samples for some species, with a total of <20 samples for *P. plunketi* and *C. owstoni*, and none for *Oxynotus bruniensis* and *Squalus griffini*. Yano & Tanaka [56] examined a large sample of *C. owstoni* (*n* = 336) and reported pelagic and benthopelagic fishes and squids, but their quantitative data were combined with *C. coelolepis* (*n* = 64) for analyses, and therefore their data were excluded from this review. *Squalus acanthias* was well studied, and the review for this species was not exhaustive.

**Table 6:** Summary of Squaliforme shark diet studies for species commonly caught during Chatham Rise bottom trawl surveys.

Species	Length (cm)	Region	*n*	Diet	Reference	Statistic	Trophic level
*Centrophorus squamosus*	160	New Zealand	26	Benthic, demersal, and benthopelagic fishes, including scavenging.	[11]	%W	4.27
		North Atlantic	21	Demersal fishes, some cephalopods.	[30]	Occurrence	–
		Southern Africa	18	Mainly cephalopods, teleosts, and some crustaceans.	[29]	%W	4.22
		Southern Africa	Not specified	Mesopelagic fishes and pelagic cephalopods	[31]	Qualitative	–
*Centroscymnus owstoni*	120	New Zealand	19	Mesopelagic and benthopelagic teleosts, with some cephalopods, crustaceans, and salps.	[11]	%W	4.24
		Australia	2	Benthopelagic teleosts, with some salps.	[32]	%W	4.19
		Australia	Not specified	Fish and cephalopods	[8]	Qualitative	–
*Centroselachus crepidater*	105	New Zealand	19	Mesopelagic teleosts, some cephalopods and crustaceans	[11]	%W	4.24
		Australia	43	Bathypelagic and mesopelagic teleosts, some cephalopods.	[32]	%W	4.24
		Australia	31	Mainly mesopelagic and bathypelagic teleosts, cephalopods, some crustaceans, and mammals.	[33]	%W	4.23
		Australia	Not specified	Fish and cephalopods	[8]	Qualitative	–
		North Atlantic	97	Cephalopods and mesopelagic teleosts, some crustaceans	[30]	%O	–
		Southern Africa	4	Mesopelagic teleosts.	[29]	%N	4.24
		Southern Africa	Not specified	Myctophids and pelagic cephalopods.	[31]	Qualitative	–
*Dalatias licha*	160	New Zealand	19	Predominantly benthopelagic fishes, including chunks of flesh.	[11]	%W	4.52
		Aegean Sea	2	Almost entirely cephalopods, traces of fishes and crustaceans.	[34]	%W	4.20
		Australia	5	Predominantly bathypelagic teleosts, some cephalopods, crustaceans, and mammals.	[33]	%N	4.44
		Australia	Not specified	Mainly teleosts, also elasmobranchs, cephalopods and crustaceans. Often chunks of flesh.	[8]	Qualitative	–
		Mediterranean Sea	31 (total)	Fishes, with some cephalopods and natant decapods.	[35]	Semi-quantitative	–
		Not specified	Not specified	Primarily mesopelagic and bathypelagic teleosts, but also elasmobranchs, cephalopods, various invertebrates, likely scavenging, and including chunks of flesh.	[36]	Qualitative	–
*Deania calcea*	120	New Zealand	133	Predominantly mesopelagic and benthopelagic fishes, some cephalopods and natant decapods	This study	%W	4.23
		Australia	10	Almost entirely mesopelagic teleosts, some cephalopods.	[32]	%W	4.24
		Australia	18	Mainly mesopelagic teleosts, bathypelagic cephalopods, some natant decapods.	[33]	%N	4.11
		Australia	27	Almost entirely mesopelagic and pelagic teleosts.	[37]	%O	–
		Australia	Not specified	Fish (mainly myctophids), cephalopods and crustaceans	[8]	Qualitative	–
		North Atlantic	66	Mainly mesopelagic teleosts, some demersal teleosts, cephalopods, and natant decapods.	[30]	%N	4.23
		North Atlantic	29	Almost entirely mesopelagic and pelagic teleosts and cephalopods.	[38]	%V	4.23
		Southern Africa	62	Mainly mesopelagic teleosts, some cephalopods and crustaceans.	[29]	%W	4.23
		Southern Africa	Not specified	Myctophids and pelagic cephalopods	[31]	Qualitative	–
		Not specified	Not specified	Mesopelagic fish and natant decapods.	[36]	Qualitative	–
*Etmopterus baxteri*	85	New Zealand	117	Principally fish, some cephalopods.	[39]	Semi-quantitative	–
		New Zealand	25	Largely teleosts and cephalopods, some mysids and decapod crustaceans.	[40]	%W	4.21
		Australia	27	Largely benthopelagic teleosts, some cephalopods and crustaceans.	[32]	%W	4.27
		Australia	31	Bathypelagic and mesopelagic fishes, cephalopods, and some curstaceans.	[33]	%N	4.15
		Australia	113	Benthopelagic teleosts, with cephalopods, some crustaceans, and other invertebrates.	[41]	%W	4.19
*Etmopterus lucifer*	45	Australia	Not specified	Squid, teleosts (mainly myctophids) and crustaceans.	[8]	Qualitative	–
		Japan	611 (total)	Mostly mesopelagic squids, with some myctophids and euphausiids.	[42]	%N	4.18
		Southern Africa	Not specified	Myctophids and pelagic cephalopods	[31]	Qualitative	–
*Oxynotus bruniensis*	70	None	–	–	**–**	–	–
*Proscymnodon plunketi*	170	New Zealand	12	Demersal fishes, likely scavenging; no crustaceans	[11]	%W	4.30
		New Zealand	6	Teleosts and cephalopods.	[43]	Qualitative	–
		Australia	5	Fishes, cephalopods, and mammal flesh.	[33]	%N	4.34
		Australia	Not specified	Fish and cephalopods	[8]	Qualitative	–
		Not specified	Not specified	Cephalopods and teleosts.	[36]	Qualitative	–
*Squalus acanthias*	110	New Zealand	295	Mainly teleosts (benthic to pelagic), scavenging, some salps, crustaceans, cephalopods, and elasmobranchs.	This study	%W	4.20
		New Zealand	5149	Predominantly pelagic crustaceans, some fishes, salps, and cephalopods.	[44]	%N	3.55
		Australia	21	Mainly benthopelagic and pelagic teleosts, with cephalopods and crustaceans.	[33]	%N	4.12
		Black Sea	328	Mainly demersal and pelagic teleosts, some crustaceans, nematodes, and actinarians.	[45]	%O	–
		Black Sea	112	Almost entirely pelagic teleosts, some demersal fishes, crustaceans, cephalopods, and mammal flesh.	[46]	%W	4.22
		Irish Sea	435	Mainly pelagic, demersal, and benthic teleosts, with crustaceans, ctenophores, and cephalopods.	[47]	%V	4.12
		Japan	26	Almost entirely pelagic teleosts, some cephalopods and invertebrates.	[48]	%W	4.23
		Northeast Pacific	3126	Incomplete diet description. Mainly pelagic teleosts and euphausiids.	[49]	–	–
		Northwest Atlantic	1390	Mainly teleosts, and then cephalopods, bivalves and crustaceans.	[50]	%W	4.08^1^
		Northwest Atlantic	3795	Predominantly demersal and pelagic teleosts, Ctenophores, but spatially and temporally variable.	[51]	Semi-quantitative	–
		Northwest Atlantic	Not specified (*n*>20)	Sharks <61 cm: primarily cephalopods and fishes, with ctenophores; >60 cm: fishes and some cephalopods.	[52]	Semi-quantitative	–
		Southern Africa	121	Mainly mesopelagic and bathypelagic fishes, some cephalopods, and a few invertebrates.	[29]	%W	4.25
		Southwest Atlantic	2214	1980s: mainly fishes, then cephalopods and invertebrates. 1990s: teleosts and cephalopods, then crustaceans and medusae, salps and ctenophores. 2000s: mainly cephalopods, then teleosts, and crustaceans.	[53]	%W	24.20, 4.13, 4.19
		Southwest Atlantic	223	Teleosts, then ctenophores, cephalopods, scavenging.	[54]	%N	3.95^3^
		Southwest Atlantic	120	Mainly cephalopods, demersal and pelagic teleosts, and some ctenophores and other invertebrates.	[24]	%W	4.20
*Squalus griffini*	110	None	–	–	–	–	–

Unless specified otherwise, *n* is the number of stomachs that contained prey and so yielded diet information. Length is the approximate maximum total length, from McMillan et al. [55]. The term *fish* means both teleosts and elasmobranchs. The statistics are %N, percentage number; %W, percentage mass; %N, percentage number;%V, percentage volume; %O, percentage occurrence; Occurrence, presence absence and could not estimate %O; semi-quantitative, quantitative diet description but data not presented in detail (only in figures).

1 , numerous estimates were possible by area and year, but the estimated trophic level did not vary much from the estimated reported here because the diet variation was largely in the proportions of cephalopods and fish, which have similar trophic levels.

2 , estimates for 1980s, 1990s, and 2000s.

3 , estimates were possible for two size classes, but only one trophic level is reported as there was only 0.01 difference between the two.

The diets reported for each species were categorised on the basis of their diet, being most often (I) mesopelagic fishes and invertebrates (*C. crepidater*, *D. calcea*, and *E. lucifer*), (II) mesopelagic and benthopelagic fishes and invertebrates (*C. owstoni*, *E. baxteri*), (III) demersal and benthopelagic fishes (*C. squamosus*, *D. licha*, and *P. plunketi*), and (IV) generalist diet of fishes and invertebrates (*S. acanthias*). Invertebrate prey were typically numerically dominated by crustaceans, with cephalopods less frequent and numerous but relatively important by weight. Within (III), *D. licha* and *P. plunketi* was noted for consuming chunks of flesh. The lowest weighted mean trophic level was estimated for *S. acanthias* (3.84), followed by *E. lucifer* (4.18), *E. baxteri* (4.20), *D. calcea* (4.22), *C. owstoni* (4.23), *C. crepidater* (4.24), *C. squamosus* (4.24), *P. plunketi* (4.32) and *D. licha* (4.48) (Table 6).

### Distribution analyses

**Figure 3:**
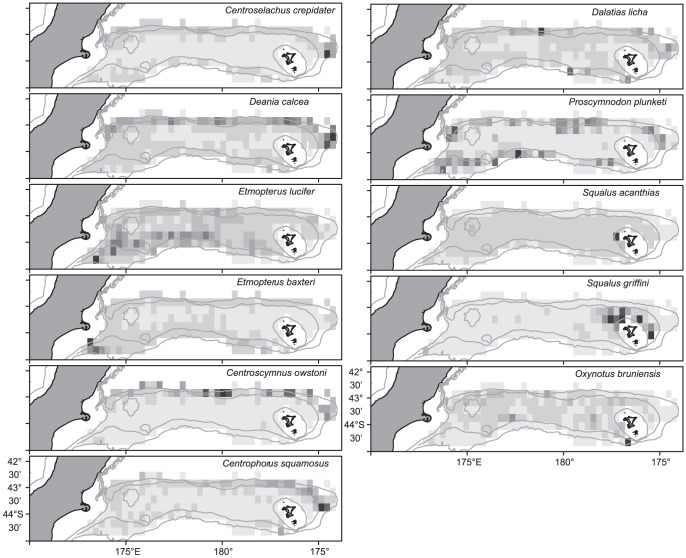
Catch rate (median kg km^−2^) of squaliforme sharks on Chatham Rise in 0.3° latitude and longitude cells. Cells shaded in the lightest grey were sampled but no catches of that species were made; cells shaded in successively darker grey had higher mean catch rates. Maximum mean catch rates: *Centroselachus crepidater* 686 kg km^−2^; *Deania calcea* 473 kg km^−2^; *Etmopterus lucifer* 12 kg km^−2^; *Etmopterus baxteri* 560 kg km^−2^; *Centroscymnus owstoni* 98 kg km^−2^; *Centrophorus squamosus* 100 kg km^−2^; *Dalatias licha* 52 kg km^−2^; *Proscymnodon plunketi* 13 kg km^−2^; *Squalus acanthias* 4630 kg km^−2^; *Squalus griffini* 10 kg km^−2^; *Oxynotus bruniensis* 9 kg km^−2^. Grey lines show the 200 m, 600 m, and 1000 m isobaths.

**Figure 4:**
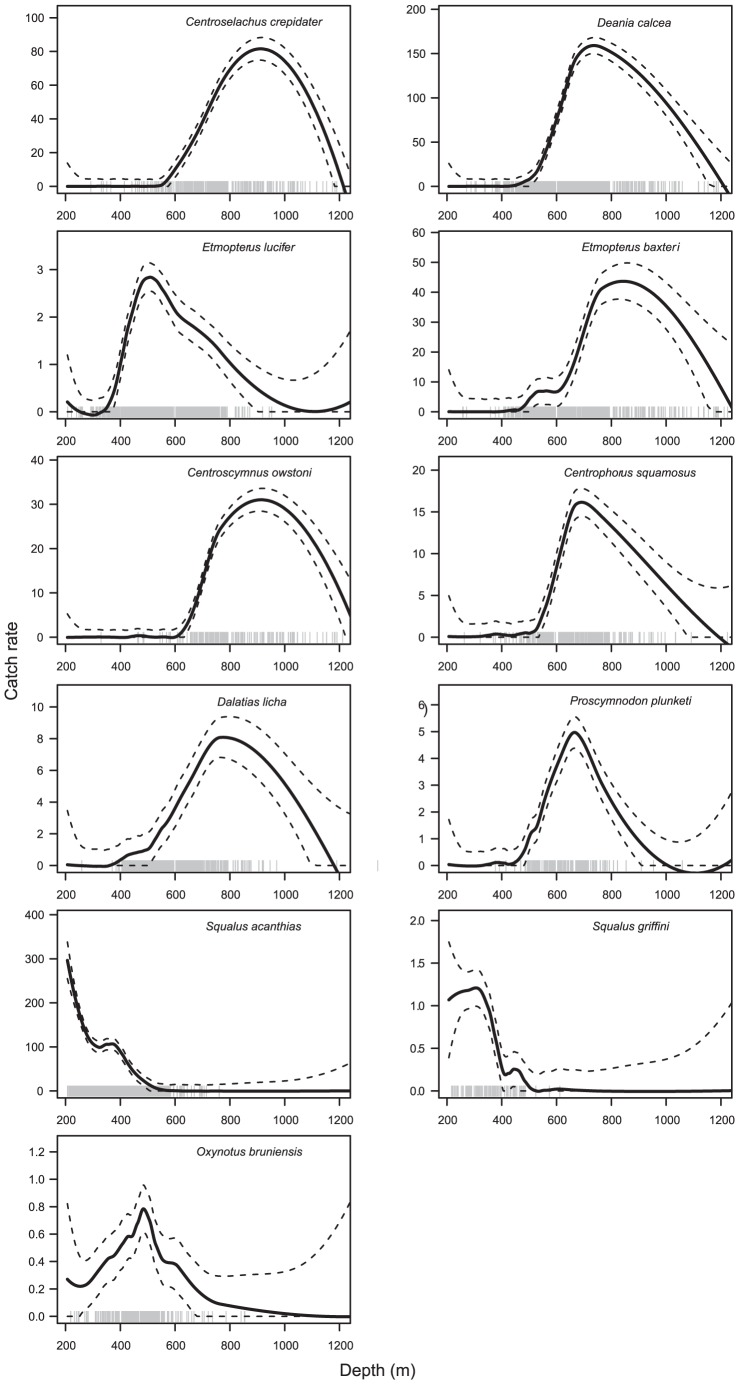
Catch rate (kg km^−2^) of squaliforme sharks on Chatham Rise by species and depth. The solid line shows the LOESS regression fitted to catch rate; broken lines indicate the 95% confidence intervals. The vertical lines above the x-axis indicate the location of catches of that species.

Amongst the sharks in diet group (I), *E. lucifer* (number of catches, *n* = 1422) was relatively ubiquitous but most abundant on the south Chatham Rise, particularly west of 180° (Fig. 3), and had a peak catch rate at about 500 m depth (Fig. 4). *Deania calcea* (*n* = 892) was most abundant along the north Chatham Rise and in particular to the north and east of the Chatham Islands, with a peak catch rate in deeper water, at 750 m. *Centroselachus crepidater* (*n* = 321) was also most abundant along the north Chatham Rise, and in particular to the east of the Chatham Islands, and the deepest peak catch rate, at about 900 m.

In diet group (II), *C. owstoni* (*n* = 177) and *E. baxteri* (*n* = 485) had similar depth distributions, with peak catch rates at about 900–1000 m, but *E. baxteri* was most abundant on the south Chatham Rise, and especially to the south and west of the Mernoo Gap, and *C. owstoni* was abundant only on the north Chatham Rise.

In diet group (III), all three species had an overlapping depth ranges with peaks in catch rate at 700–800 m, but *D. licha* (*n* = 643) had a relatively ubiquitous distribution but with persistently higher catch rates northeast of the Chatham Islands, *P. plunketi* (*n* = 186) had a patchier distribution, but was persistently more abundant on the south west and central northern Chatham Rise, and *C. squamosus* (*n* = 273) was abundant only to the north and east of the Chatham Islands. *Proscymnodon plunketi* had the narrowest depth range, with most catches made between 500–800 m, compared to 400–900 m in *D. licha*, and 400–1000 m in *C. squamosus*.

In diet group (IV), *S. acanthias* (*n* = 1520) catch rates were greatest at the shallow boundary of the survey (200 m), and then declined with depth, being rare below 500 m. *Squalus acanthias* were relatively ubiquitous, but large catch rates were taken in several years to the west of the Chatham Islands. *Squalus acanthias* had the highest catch rates in this study. *Squalus griffini* (*n* = 106) had a depth distribution broadly similar to *S. acanthias*, except that the catch rate did not increase at depths <300 m, and it was caught almost entirely around the Chatham Islands. *Oxynotus bruniensis* (*n* = 222) was relatively ubiquitous across Chatham Rise, with a peak in catch rate at about 500 m.

## Discussion

The eleven squaliforme shark species studied on Chatham Rise showed a variety of different diets, and depth and location preferences, consistent with niche separation to reduce interspecific competition. We described four trophic groups, and within these groups there was some spatial and depth separation of species. Some studies have already suggested that deep-sea sharks may reduce interspecific competition by having different prey preferences (often used to infer foraging depth), and/or different bottom depth preferences [10], [29], [30], [33], [38], [42], [57]. The only previous study of the distribution of deep water sharks on Chatham Rise [1] used catch data from bottom trawl surveys at 700–1500 m depth, and concluded there were no interspecific differences in depth distribution; all sharks were rare below 1200 m, and all extended shallower than 700 m. The depth range we studied, 200–1200 m, was more appropriate for studying shark distribution, but still did not include the shallow depth limits for *S. acanthias*, *S. griffini*, and possibly *O. bruniensis*. The data set we analysed was also more spatially extensive than Wetherbee's [1], and allowed us to identify more detailed spatial differences in distribution. In some cases the spatial separation we found was pronounced. For example, there was relatively little spatial overlap between the mesopelagic and benthopelagic predators *C. owstoni* and *E. baxteri*. Wetherbee [1] also noted *E. baxteri* was more abundant on the south Chatham Rise, and *C. owstoni* more abundant on the north.

Bulman et al. [32] listed *C. owstoni* and *E. baxteri* together with *C. crepidater* and *D. calcea* as “pelagic and benthopelagic piscivores”. We agree with the grouping of *C. owstoni* and *E. baxteri*, but suggest these two species are usually more benthopelagic, and *C. crepidater* and *D. calcea* are usually more mesopelagic. Amongst the predators of mesopelagic fishes and invertebrates, the small (for a shark) *E. lucifer* was shallower and more southern in distribution than both *C. crepidater* and *D. calcea*, but the spatial overlap between the latter two species was relatively high, with both species especially abundant to the east of the Chatham Islands. Whilst *C. crepidater* did tend to occur a little deeper than *D. calcea*, there could be separation between *C. crepidater* and *D. calcea* to reduce competition that was not apparent from depth and location, for example the two species might forage at different depths in the mesopelagic layers, or at different times of day. Competition between all three predators of mesopelagic fishes and invertebrates might also be reduced by the relatively high abundance of mesopelagic prey on Chatham Rise [17], [37]. Off southern Africa, Macpherson & Roel [31] similarly classified *E. lucifer*, *C. crepidater* and *D. calcea* together as foraging on “myctophids and pelagic cephalopods”, but also included *C. squamosus* in this group. The former three species, although variable in size and in different genera, all have relatively slender bodies and flattened heads and snouts (they have a “shovelnose” dogfish appearance). *Centrophorus squamosus* has a stockier body and more rounded snout, and on the basis of diet we classified *C. squamosus* with the similarly large, relatively stocky, and blunt snouted *D. licha*, and *P. plunketi*.

*Centrophorus squamosus* had a relatively distinct distribution on Chatham Rise, but there was more general overlap between the distributions of *D. licha*, and *P. plunketi*, although the high density areas for each species tended not to overlap. However, both *D. licha*, and *P. plunketi* had relatively low average catch rates given their relatively large size, indicating a relatively low abundance, which may reduce competition. It is unclear whether the relatively low abundance of *D. licha* and *P. plunketi* on Chatham Rise is natural or a consequence of overfishing. *Proscymnodon plunketi* biomass apparently declined in bottom trawl surveys of the orange roughy spawning grounds (depths of about 800–1200 m) on the northeast Chatham Rise, with biomass in 1994 just 6% of that in 1984 [58]. Subsequent, more extensive trawl surveys, although relatively poor in quality for *P. plunketi* (relatively high coefficients of variation), indicate abundance did not change substantially between 1992 and 2010 [9]. Although the two trawl survey series are not strictly comparable, the biomass trend they could indicate is a large decline in *P. plunketi* during the 1980s and early 1990s, followed by a low but stable biomass level since then. In which case, fishing may have modified the degree of resource competition, and possibly diet and distribution, of *P. plunketi* and *D. licha*.

*Squalus acanthias* and *S. griffini* do not have the dark colouration typical of many deepsea sharks, and morphologically they are very alike. *Squalus acanthias* has been studied worldwide, with the diet often described as “opportunistic” although usually dominated by pelagic fishes. Although there are no diet data for *S. griffini*, it seems likely that the two species have similar habits, with *S. griffini* being the subtropical congener of the temperate *S. acanthias*.

Very little is known about *Oxynotus bruniensis*, but its relatively small ventral mouth would suggest it eats small benthic prey. The diet of *Oxynotus centrina* in the north Atlantic was found to consist of predominantly benthic worms (Polychaeta and Sipunculidae), with some crustaceans, teleosts, and echinoderms [59]. The diet of *Oxynotus bruniensis* could therefore have greater similarity to chimaeras and the smaller skates and rays [20], [60].

The trophic levels estimated for the sharks in diet groups I (*C. crepidater*, *D. calcea*, and *E. lucifer*) and II (*C. owstoni*, *E. baxteri*) were similar, reflecting a broadly similar diet of predominantly teleosts and cephalopods. The trophic levels of the relatively large *D. licha* and *P. plunketi* were slightly higher, primarily because they predated other elasmobranchs, and also chunks of mammal flesh. The latter may indicate these species feed like cookie cutter sharks (*Isistius* spp.) [9]. In the similarly large *C. squamosus*, elasmobranchs were less frequent in the diet, and mammal flesh absent, and the trophic level was accordingly lower (4.24). The trophic level of *S. acanthias* was the lowest, although strongly influenced by the crustacean-focused diet reported in a large sample by Hanchet [44]. If Hanchet's [44] study was removed, the mean weighted trophic level for *S. acanthias* was higher (4.13), although still the lowest of the species studied here.

The trophic levels estimated here were the same as estimated by Cortés [22] for *C. crepidater*, *C. squamosus*, *D. calcea*, and *E. baxteri* (4.2). Cortés [22] estimated the trophic level of *E. lucifer* to be a little lower (4.1) than the present study (4.18). *Squalus acanthias* had the lowest trophic level, with Cortés [22] estimating 3.9 and the present study 3.84. Whilst we estimated a relatively high trophic level for *D. licha* (4.48), Cortés [22] estimated a relatively low trophic level (4.1) from a reported diet of teleosts, crustaceans, elasmobranchs, cephalopods, and other invertebrates. The relatively large size of *D. licha*, and frequent reports of other elasmobranchs in the diet, suggest this species should have a relatively high trophic level (at least as an adult). Cortés [22] did not report trophic level estimates for *C. owstoni*, *P. plunketi*, *S. griffini* or *O. bruniensis*.

Our conclusions about diet and resource partitioning of squaliforme sharks could easily be biased by small sample sizes (*n*<50), which are typical for diet studies of deep sea sharks (see Table 6). Our samples of *S. acanthias* demonstrated the potential for bias: whilst we concluded that the diet of *S. acanthias* on Chatham Rise was varied but predominantly fishes, had we sampled only in 2006 we would have concluded that the diet was predominantly cephalopods. The research trawl survey biomass estimates for *Nototodarus sloanii*, the commonest squid caught during the survey on Chatham Rise, suggested that in 2006 *N. sloanii* were at least five times more abundant than in 2005 or 2007 [9]. This correlation between *S. acanthias* diet and the relative high abundance of trawl caught squid in 2006 suggests *S. acanthias* has adaptive foraging. It may be that the diets of many deep water sharks are more diverse than existing samples suggest. For example, the apparent absence of crustacean prey in the diet of *C. squamosus* and *P. plunketi* on Chatham Rise could easily be a bias caused by small sample sizes. Although stomach contents analyses do have several potential biases, they are a widely-used and accepted method that can provide information on a species role in food webs [61], and are complementary with other methods [62]. In this study, despite the potential for bias caused by small sample sizes, and by spatially and temporally diverse sampling locations, the diets reported for most deep water sharks were reasonably consistent worldwide.

Although we classified the sharks into four diet groups, the prey were not exclusive, and it seems likely that all species show some dietary overlap and adaptive foraging. Fatty acid signatures from myctophid prey have been identified in several sympatric deepwater sharks, including *S. acanthias*, *C. crepidater*, *D. licha*, *P. plunketi*, *C. owstoni*, *D. calcea*, and *E. baxteri* [33]. Scavenging of natural food fall, or of discarded offal from fishing vessels, has also been reported or suspected in a variety of deep-sea sharks [11], [32], [41], although the difficulty in identifying scavenged prey means it may be more widespread and important than currently thought. This also means that local fishing practices may bias shark diet, and from this the interpretations of foraging behaviour. The conclusions from our study are therefore contingent on our samples, and the degree of dietary and distributional overlap may well vary with time and location, and potentially other biological factors such as ontogeny.

The diets we estimated for *D. calcea* and *S. acanthias* on Chatham Rise were similar to that reported elsewhere, with *D. calcea* primarily a mesopelagic piscivore, and *S. acanthias* primarily an adaptive piscivore. All reports of *D. calcea* off New Zealand, Australia, and southern Africa indicated a diet dominated by pelagic fishes such as myctophids and mackerels (Carangidae) with relatively low prey diversity [29], [32], [33], [37]. In the North Atlantic, mesopelagic prey did feature, but there were higher proportions of demersal fishes in the diet [30], [38]. Although sample sizes were relatively small and discrete, there appear to be no obvious differences in reported sample characteristics (e.g., season, depth, fish size) that might explain the difference in diet, so it could well be related to location and local prey availability. Therefore, although *D. calcea* may specialise on mesopelagic prey, it apparently retains some ability to forage adaptively.

The diet of *S. acanthias* on Chatham Rise was characterised by fishes, although the commonest fish prey were suspected to be scavenged offal from fishing vessels. Compared to other sharks, *S. acanthias* reportedly has an exceptionally adaptive or “opportunistic” foraging behaviour, a conclusion supported by substantial spatial and temporal variations in diet [24], [46], [53], [54]. Whilst adaptive foraging could potentially mask diet changes with ontogeny, we found smaller sharks eating notably more small crustaceans and salps, and larger sharks more large and scavenged fishes. Whilst most other studies [24], [47], [49], [54], but not all [45], [53], have reported similar ontogentic shifts in diet, some form of diet change with size is expected [63]. The study of *S. acanthias* off the east coast of the South Island of New Zealand appears to have reported an exceptional diet, being dominated by crustaceans instead of fishes, and including cannabilism [44]. The relative availability of different potential prey for *S. acanthias* on Chatham Rise (this study) and the east coast South Island [44] is unknown. Whilst differences in diet could well reflect real persistent regional differences, such differences could easily be confused by variable prey availability and restricted sampling if combined with pronounced adaptive foraging. Pronounced adaptive foraging may make a species relatively resilient to fisheries-induced ecosystem change, and accordingly *S. acanthias* was the only squaliforme shark to increase in abundance during research trawl surveys between 1992 and 2010 [9].

Fisheries may affect deep-sea shark populations in two main ways. First, capture in nets causes fishing mortality, and escape from nets may result in behavioural impairment and subsequent natural mortality [64]. Second, fishing may influence population productivity, or natural mortality, through the modification of habitats and resources. The sharks foraging on mesopelagic and benthopelagic fishes are directly competing for food resources with hoki, the most abundant species in bottom trawl surveys [9], and the most important commercial fish stock on Chatham Rise [4]. Although the hoki stock has been depleted by fishing to about 50% of its original size [4], the resulting reduction in competition has apparently not resulted in a net benefit to sharks foraging on mesopelagic and benthopelagic resources; the research survey biomass trends for *C. crepidater*, *D. calcea*, and *E. lucifer* have all shown no trend in population size between 1992 and 2010 [9]. It may be that increased mortality from fishing compensates for any decrease in competition. On the east coast of the North Island of New Zealand, research trawl surveys over 600–1500 m and between 1992–94 and 2010 showed a significant increase in biomass of *E. lucifer*, no change in *D. calcea*, and a significant decrease in *C. crepidater* [65]. *Etmopterus lucifer* may be the species most likely to benefit from reduced competition with hoki, because its small size means it could most readily escape trawl nets, and so suffer relatively low fishing mortality.

In principle, the greater the association a shark has with the sea bed the more vulnerable it may be to bottom trawling. The species foraging primarily on mesopelagic prey must spend part of their time in mid-water, where they are not vulnerable to bottom trawls. Demersal foraging shark species in greatest abundance on the west and northwest Chatham Rise, where bottom trawl effort is focused [66], may therefore be at greatest risk from fishing mortality. Assuming that there is not movement of sharks outside of Chatham Rise, the shark most at risk would probably be *P. plunketi*, followed by *O. bruniensis*, *D. licha*, and then to a lesser extent *E. baxteri*, *C. owstoni*, and *S. acanthias*. Although predominantly a demersal species, the north-eastern distribution of *C. squamosus* would make it lower risk. However, none of these species have shown a strong biomass trend in research trawl surveys between 1992 and 2010 [9]. *Proscymnodon plunketi* biomass apparently declined on the northeast Chatham Rise between 1984 and 1994 [57], and in the same surveys, *E. baxteri* biomass also decreased (to 26% in 1994), but *C. owstoni*, *C. crepidater*, and *D. calcea* biomass increased.

Demersal foraging sharks would probably have greatest competition for resources with large and relatively abundant piscivorous bony fishes such as hake *Merluccius australis* and ling *Genypterus blacodes* [67]. Both hake and ling are targeted by commercial fisheries on Chatham Rise [4]. The ling has been found to consume substantial amounts of scavenged offal, most likely discards from fishing vessels [67], and it seems likely that benthic skates [60] and demersal sharks do the same [11]. The increase in the availability of scavenged prey may provide a positive feedback to shark productivity, which may compensate, to some extent, for the increase in fishing mortality. Sharks may also benefit from predating behaviourally impaired fish, of many species, that have escaped trawl nets [64]. Changes to fishing regulations and fishing practices, in order to reduce by-catch and discards, could therefore have a negative effect on the food supply, and therefore productivity, of demersal foraging sharks.

## Supporting Information

Table S1 **Stomach contents composition for** ***Deania calcea*** **from the Chatham Rise 2005, 2006 and 2007 combined.**
(DOCX)

Table S2 **Stomach contents composition for** ***Squalus acanthias*** **from the Chatham Rise 2005, 2006 and 2007 combined.**
(DOCX)

## References

[1] Wetherbee BM (2000) Assemblage of deep-sea sharks on Chatham Rise, New Zealand. Fish Bull 98: 189–198.

[2] Blackwell RG (2010) Distribution and abundance of deepwater sharks in New Zealand waters, 2000–01 to 2005–06. N Z Aquat Environ Biodiversity Rep 57. Wellington: Ministry for Primary Industries.

[3] White WT, Blaber SJM, Craig JF (2012) The current status of elasmobranchs: biology, fisheries and conservation. J Fish Biol 80: 897–900. 22497366 10.1111/j.1095-8649.2012.03268.x

[4] Ministry for Primary Industries (2012) Report from the Fisheries Assessment Plenary, May 2012: stock assessments and yield estimates. Compiled by the Fisheries Science Group, Ministry for Primary Industries, Wellington, New Zealand. Wellington: Ministry for Primary Industries.

[5] Francis MP (1998) New Zealand shark fisheries: development, size and management. Mar Fresh Res 49: 579–591.

[6] Graham KJ, Andrew NL, Hodgson KE (2001) Changes in relative abundance of sharks and rays on Australian South East Fishery trawl grounds after twenty years of fishing. Mar Fresh Res 52: 549–561.

[7] Stevens JD, Bonfil R, Dulvy NK, Walker PA (2000) The effects of fishing on sharks, rays, and chimaeras (chondrichthyans), and implications for marine ecosystems. ICES J Mar Sci 57: 476–494.

[8] Last PR, Stevens JD (2009) Sharks and Rays of Australia second edition. CSIRO Publishing, Collingwood, Australia.

[9] O'Driscoll RL, MacGibbon D, Fu D, Lyon W, Stevens DW (2011) A review of hoki and middle depth trawl surveys of the Chatham Rise, January 1992–2010. N Z Fish Assess Rep 2011/47. Wellington: Ministry for Primary Industries.

[10] Livingston ME (1990) Spawning hoki (*Macruronus novaezelandiae* Hector) concentrations in Cook Strait and off the east coast of the South Island, New Zealand, August–September 1987. N Z J Mar Fresh Res 24: 503–517.

[11] Dunn MR, Szabo A, McVeagh M, Smith PJ (2010) The diet of deepwater sharks and the benefits of using DNA identification of prey. Deep Sea Res I 57: 923–930.

[12] Tyrrell MC, Link JS, Moustahfid H, Overholtz WJ (2008) Evaluating the effect of predation mortality on forage species population dynamics in the Northeast US continental shelf ecosystem using multispecies virtual population analysis. ICES J Mar Sci 65: 1689–1700.

[13] Moustahfid H, Link JS, Overholtz WJ, Tyrrell MC (2009) The advantage of explicitly incorporating predation mortality into age-structured stock assessment models: an application for Atlantic mackerel. ICES J Mar Sci 66: 445–454.

[14] Rooney N, McCann KS (2012) Integrating food web diversity, structure and stability. Trends Ecol Evol 27: 40–46. 21944861 10.1016/j.tree.2011.09.001

[15] Ministry for Primary Industries (2008) National plan of action for sharks. Available from: http://www.fish.govt.nz/NR/rdonlyres/F0530841-CD61-4C3E-9E50-153A281A4180/0/NPOAsharks.pdf Accessed 28 June 2012.

[16] Murphy RJ, Pinkerton MH, Richardson KM, Bradford-Grieve JM, Boyd PW (2001) Phytoplankton distributions around New Zealand derived from SeaWiFS remotely-sensed ocean colour data. N Z J Mar Fresh Res 35: 343–362.

[17] McClatchie S, Dunford A (2003) Estimated biomass of vertically migrating mesopelagic fish off New Zealand. Deep-Sea Res I 50: 1263–1281.

[18] Connell A, Dunn MR, Forman J (2010) Diet and dietary variation of New Zealand hoki *Macruronus novaezelandiae* . N Z J Mar Fresh Res 44: 289–308.

[19] Stevens DW, Dunn MR (2011) Different food preferences in four sympatric deep-sea Macrourid fishes. Mar Biol 158: 59–72.

[20] Dunn MR, Griggs L, Forman J, Horn P (2010) Feeding habits and niche separation among the deep-sea chimaeroid fishes *Harriotta raleighana*, *Hydrolagus bemisi* and *Hydrolagus novaezealandiae* . Mar Ecol Prog Ser 407: 209–225.

[21] Pinkas L, Oliphant MS, Iverson LK (1971) Food habits of albacore, bluefin tuna, and bonito in California waters. Fish Bull Calif Dept Fish Game 152.

[22] Cortés E (1997) A critical review of methods of studying fish feeding based on analysis of stomach contents: application to elasmobranch fishes. Can J Fish Aquat Sci 54: 726–738.

[23] Tirasin EM, Jørgensen T (1999) An evaluation of the precision of diet description. Mar Ecol Prog Ser 182: 243–252.

[24] Koen Alonso M, Crespo AE, García AN, Pedraza NS, Mariotti AP, et al (2002) Fishery and Ontogenetic Driven Changes in the Diet of the Spiny Dogfish, *Squalus acanthias*, in Patagonian Waters, Argentina. Environ Biol Fishes 63: 193–202.

[25] Dunn MR (2009) Feeding habits of the ommastrephid squid *Nototodarus sloanii* on the Chatham Rise, New Zealand. N Z J Mar Freshw Res 43: 1103–1113.

[26] Anderson MJ, Gorley RN, Clarke KR (2008) PERMANOVA+ for PRIMER: guide to software and statistical methods. Plymouth: PRIMER-E.

[27] Kuha J (2004) AIC and BIC: Comparisons of assumptions and performance. Sociol Meth Res 33: 188–229.

[28] Clarke KR, Gorley RN (2006) PRIMER v6: User Manual/Tutorial. Plymouth: PRIMER-E.

[29] Ebert DA, Compagno LJV, Cowley PD (1992) A preliminary investigation of the feeding ecology of squaloid sharks off the west coast of southern Africa. Benguela Trophic Functioning. S Afr J Mar Sci 12: 601–609.

[30] Mauchline J, Gordon JDM (1983) Diets of the sharks and chimaeroids of the Rockall Trough, northeastern Atlantic Ocean. Mar Biol 75: 269–278.

[31] Macpherson E, Roel BA (1987) Trophic relationships in demersal fish community off Namibia. S Afr J Mar Sci 5: 585–596.

[32] Bulman CM, He X, Koslow JA (2002) Trophic ecology of the mid-slope demersal fish community off southern Tasmania, Australia. Mar Fresh Res 53: 59–72.

[33] Pethybridge H, Daley RK, Nichols PD (2011) Diet of demersal sharks and chimaeras inferred by fatty acid profiles and stomach content analysis. J Exp Mar Biol Ecol 409: 290–299.

[34] Karachle PK, Stergiou KI (2010) Food and feeding habits of nine elasmobranch species in the N Aegean Sea. Rapp Comm int Mer Médit 39: 553.

[35] Macpherson E (1979) Relations Trophiques dea poissons dans la Mediterranee occidentale. Rapports et proces-verbaux des reunions Commission internationale pour l'exploration scientifique de la Mer Méditerranee 25–26: 49–57.

[36] Compagno LJV (1984) FAO Species Catalogue. Vol. 4. Sharks of the world. An annotated and illustrated catalogue of shark species known to date. Part 1 - Hexanchiformes to Lamniformes. FAO Fish Synop 125 ((4/1)) 1–249 Rome: FAO.

[37] Blaber SJM, Bulman CM (1987) Diets of fishes of the upper continental slope of eastern Tasmania: content, calorific values, dietary overlap and trophic relationships. Mar Biol 95: 345–356.

[38] Preciado I, Cartes JE, Serrano A, Velasco F, Olaso I, et al (2009) Resource utilization by deep-sea sharks at the Le Danois Bank, Cantabrian Sea, north-east Atlantic Ocean. J Fish Biol 75: 1331–1355. 20738618 10.1111/j.1095-8649.2009.02367.x

[39] Clark MR, King KJ, McMillan PJ (1989) The food and feeding relationships of black oreo, *Allocyttus niger*, smooth oreo, *Pseudocyttus maculatus*, and eight other fish species from the continental slope of the south-west Chatham Rise, New Zealand. J Fish Biol 35: 465–484.

[40] Jones MRL (2008) Biology and diet of *Coryphaenoides subserrulatus* and *Etmopterus baxteri* from the Puysegur region, southern New Zealand. N Z J Mar Fresh Res 42: 333–337.

[41] Hallet CS, Daley RK (2011) Feeding ecology of the southern lanternshark (*Etmopterus baxteri*) and the brown lanternshark (*E.unicolor*) off southeastern Australia. ICES J Mar Sci 68: 157–165.

[42] Baba O, Taniuchi T, Nose Y (1987) Depth distribution and food habits of three species of small squaloid sharks off Choshi. Nippon Suisan Gakkasishi 53: 417–424.

[43] Garrick JAF (1959) Studies on New Zealand Elasmobranchii – Part IX. *Scymnodon plunketi* (Waite, 1910), an abundant deep-water shark of New Zealand waters. Trans Royal Soc N Z 87: 271–282.

[44] Hanchet S (1991) Diet of spiny dogfish, *Squalus acanthias* Linnaeus, on the east coast, South Island, New Zealand. J Fish Biol 39: 313–323.

[45] Avsar D (2001) Age, growth, reproduction and feeding of the spurdog (*Squalus acanthias* Linnaeus, 1758) in the south-eastern Black Sea. Estuar Coast Shelf Sci 52: 269–278.

[46] Demirhan SA, Seyhan K (2007) Life history of spiny dogfish, *Squalus acanthias* (L. 1758), in the southern Black Sea. Fish Res 85: 210–216.

[47] Ellis JR, Pawson MG, Shackley SE (1996) The comparative feeding ecology of six species of shark and four species of ray (Elasmobranchii) in the north-east Atlantic. J Mar Biol Ass UK 76: 89–106.

[48] Fujita T, Kitagawa D, Okuyama Y, Ishito Y, Inada T, et al (1995) Diets of the demersal fishes on the shelf off Iwate, northern Japan. Mar Biol 123: 219–233.

[49] Tanasichuk RW, Ware DM, Shaw W, McFarlane GA (1991) Variations in diet, daily ration, and feeding periodicity of Pacific Hake (*Merluccius productus*) and spiny dogfish (*Squalus acanthias*) off the lower west coast of Vancouver Island. Can J Fish Aquat Sci 48: 2118–2128.

[50] Bowman RE, Stillwell CE, Michaels WL, Grosslein MD (2000) Food of northwest Atlantic fishes and two common species of squid. NOAA Tech Memo NMFS-NE 155. 138 p.

[51] Bundy A, Link JS, Smith BE, Cook AM (2011) You are what you eat, whenever or wherever you eat it: an integgrative analysis of fish food habits in Canadian and U.S.A waters. J Fish Biol 78: 514–539. 21284632 10.1111/j.1095-8649.2010.02868.x

[52] Garrison LP, Link JS (2000) Dietary guild structure of the fish community in the Northeast United States continental shelf ecosystem. Mar Ecol Prog Ser 202: 231–240.

[53] Belleggia M, Figueroa DE, Sánchez F, Bremec C (2012) Long-term changes in the spiny dogfish (*Squalus acanthias*) trophic role in the southwestern Atlantic. Hydrobiologia 684: 57–67.

[54] Laptikhovsky VV, Arkhipkin AI, Henderson AC (2001) Feeding habits and dietary overlap in spiny dogfish *Squalus acanthias* (Squalidae) and narrowmouth catshark *Schroederichthys bivius* (Scyliorhinidae). J Mar Biol Ass UK 81: 1015–1018.

[55] McMillan PJ, Francis MP, James GD, Paul LJ, Marriott PJ, et al.. (2011) New Zealand fishes, Volume 1: A field guide to common species caught by bottom and midwater fishing. N Z Aquat Environ Biodiversity Rep 68. Wellington: Ministry for Primary Industries.

[56] Yano K, Tanaka S (1984) Some biological aspects of the deep sea squaloid shark Centroscymnus from Suruga Bay, Japan. Bull Jap Soc Sci Fish.Nissuishi 50: 249–256.

[57] Carrassón M, Stefanescu C, Cartes JE (1992) Diets and bathymetric distributions of two bathyal sharks of the Catalan deep sea (western Mediterranean). Mar Ecol Prog Ser 82: 21–30.

[58] Clark MR, Anderson OF, Francis RICC, Tracey DM (2000) The effects of commercial exploitation on orange roughy (*Hoplostethus atlanticus*) from the continental slope of the Chatham Rise, New Zealand, from 1979 to 1997. Fish Res 45: 217–238.

[59] Capape C (2008) Diet of the angular rough shark *Oxynotus centrina* (Chondrichthyes: Oxynotidae) off he Languedocian coast (Southern France, North-western Mediterranean). Vie et Milieu 58: 57–61.

[60] Forman JS, Dunn MR (2012) Diet and scavenging habits of the smooth skate *Dipturus innominatus* from Chatham Rise, New Zealand. J Fish Biol 80: 1546–1562. 22497396 10.1111/j.1095-8649.2012.03255.x

[61] Stergiou KI, Karpouzi VS (2002) Feeding habits and trophic levels of Mediterranean fish. Rev Fish Biol Fish 11: 217–254.

[62] Pasquaud S, Pillet M, David V, Sautour B, Elie P (2010) Determination of fish trophic levels in an estuarine system. Estuar Coast Shelf Sci 86: 237–246.

[63] Werner EE (1984) Gilliam (1984) The ontogenetic niche and species interactions in size-structured populations. Ann Rev Ecol Syst 15: 393–425.

[64] Ryer CH (2004) Laboratory evidence for behavioural impairement of fish escaping trawls: a review. ICES J Mar Sci 61: 1157–1164.

[65] Doonan IJ, Dunn MR (2011) Trawl survey for Mid-East Coast orange roughy: March–April 2010. N Z Fish Assess Rep 2011/20. Wellington: Ministry for Primary Industries.

[66] Baird SJ, Wood BA (2012) Extent of coverage of 15 environmental classes within the New Zealand EEZ by commercial trawling with seafloor contact. N Z Aquat Environ Biodiversity Rep 88. Wellington: Ministry for Primary Industries.

[67] Dunn MR, Connell AM, Forman J, Stevens D, Horn P (2010) Diet of two large sympatric teleosts, the ling (*Genypterus blacodes*) and hake (*Merluccius australis*). PLoS ONE 5 ((10)) e13647 doi:10.1371/journal.pone.0013647. 21048962 10.1371/journal.pone.0013647 PMC2965093

