# Supplementary material for: Trophic Interactions and Distribution of Some Squaliforme Sharks, Including New Diet Descriptions for Deania calcea and Squalus acanthias
Source: PLoS One. 2013 Mar 25;8(3):e59938. doi: 10.1371/journal.pone.0059938 (PMC3607562; doi:10.1371/journal.pone.0059938)
Supplement: Table S1 — Stomach contents composition for Deania calcea from the Chatham Rise 2005, 2006 and 2007 combined. (DOCX) [file pone.0059938.s001.docx]

**Table S1:** Stomach contents composition for *Deania calcea* from the Chatham Rise 2005, 2006 and 2007 combined.

|  |  |  |  |  |  |  |
| --- | --- | --- | --- | --- | --- | --- |
|  |  |  | %F | %W | %N | %IRI |
|  |  |  |  |  |  |  |
| **Salpida** | | | **3.0 (0–8.3)** | **<0.1** | **3.7 (0–10.7)** | **0.2 (0–1.8)** |
|  |  | Salpida unidentified | 1.87 | <0.01 | 1.91 | 0.09 |
| **Natant Decapoda** | | | **25.6 (15.1–35.4)** | **2.5 (0.7–6.5)** | **20.1 (11.2–29.6)** | **12.2 (3.5–24.4)** |
|  | Pasiphaeidae | |  |  |  |  |
|  |  | *Pasiphaea* spp. | 6.54 | 0.55 | 4.09 | 0.80 |
|  | Oplophoridae | |  |  |  |  |
|  |  | *Acanthephyra* spp. | 0.47 | 0.07 | 0.27 | <0.01 |
|  |  | *Oplophorus novaezeelandiae* | 1.87 | 0.36 | 1.09 | 0.07 |
|  | Sergestidae | |  |  |  |  |
|  |  | *Sergestes* spp. | 2.34 | 0.05 | 1.36 | 0.09 |
|  | Natant decapoda unidentified | | 5.61 | 0.52 | 3.54 | 0.60 |
| **Chondrichthyes** | | | **0.8 (0–2.8)** | **1.6 (0–7.4)** | **0.5 (0–2.0)** | **<0.1 (0–0.5)** |
|  | Shark unidentified | | 0.47 | 0.98 | 0.27 | 0.02 |
| **Osteichthyes** | | |  |  |  |  |
|  | **Sternoptychidae** | | **12.8 (1.6–27.5)** | **2.0 (0.1–7.4)** | **15.3 (1.2–33.4)** | **4.7 (0–23.0)** |
|  |  | *Maurolicus australis* | 7.94 | 1.25 | 7.90 | 1.92 |
|  | **Photichthyidae** | | **1.5 (0–5.2)** | **13.7 (0–47.7)** | **1.1 (0–3.9)** | **0.5 (0–5.7)** |
|  |  | *Phosichthys argenteus* | 0.93 | 8.48 | 0.54 | 0.22 |
|  | **Notosudidae** | | **0.8 (0–3.5)** | **2.2 (0–10.4)** | **0.5 (0–2.6)** | **<0.1 (0–0.9)** |
|  |  | *Scopelosaurus* spp. | 0.47 | 1.34 | 0.27 | 0.02 |
|  | **Myctophidae** | | **59.4 (46.9–70.7)** | **13.0 (5.2–30.8)** | **47.6 (36.0–59.6)** | **76.0 (53.6–85.8)** |
|  |  | *Diaphus danae* | 0.47 | 0.14 | 0.27 | 0.01 |
|  |  | *Electrona* spp. | 0.47 | 0.10 | 0.27 | <0.01 |
|  |  | *Lampanyctodes hectoris* | 14.95 | 2.75 | 10.63 | 5.28 |
|  |  | *Lampanyctus* spp. | 0.47 | 0.18 | 0.27 | 0.01 |
|  |  | *Symbolophorus* *boops* | 0.47 | 0.50 | 0.27 | 0.01 |
|  |  | Myctophidae unidentified | 20.09 | 4.39 | 12.53 | 8.98 |
|  | **Merlucciidae** | | **1.5 (0–5.4)** | **20.2 (0–53.1)** | **1.1 (0–3.9)** | **0.7 (0–5.8)** |
|  |  | *Macruronus novaezelandiae* | 0.93 | 12.5 | 0.54 | 0.32 |
|  | **Macrouridae** | | **4.5 (0.7–9.1)** | **20.0 (0.1–50.6)** | **3.2 (0.5–6.7)** | **2.2 (0–9.2)** |
|  |  | *Coelorinchus innotabilis* | 0.47 | 1.01 | 0.27 | 0.02 |
|  |  | *C. oliverianus* | 0.47 | 1.17 | 0.27 | 0.02 |
|  |  | *C.* spp. | 0.47 | <0.01 | 0.27 | <0.01 |
|  |  | *Lepidorhynchus denticulatus* | 0.93 | 9.62 | 0.54 | 0.25 |
|  |  | Macrouridae unidentified | 0.47 | 0.58 | 0.27 | 0.01 |
|  | **Trachichthyidae** | | **0.8 (0–3.4)** | **0.1 (0–0.7)** | **0.5 (0–2.5)** | **<0.1 (0–0.2)** |
|  |  | *Paratrachichthys trailli* | 0.47 | 0.09 | 0.27 | <0.01 |
|  | **Zeidae** | | **0.8 (0–3.5)** | **9.2 (0–36.0)** | **0.5 (0–2.5)** | **0.2 (0–2.6)** |
|  |  | *Cyttus novaezelandiae* | 0.47 | 5.68 | 0.27 | 0.07 |
|  | **Carangidae** | | **0.8 (0–2.8)** | **0.1 (0–0.3)** | **0.5 (0–2.0)** | **<0.1 (0–0.1)** |
|  |  | *Trachurus* spp. | 0.47 | 0.04 | 0.27 | <0.01 |
| **Cephalopoda** | | |  |  |  |  |
| **Teuthoidea** | | | **7.5 (2.1–14.8)** | **15.4 (0.9–42.1)** | **5.3 (1.4–10.9)** | **3.3 (0.2–13.2)** |
|  | Onychoteuthidae | |  |  |  |  |
|  |  | *Onykia ingens* | 1.40 | 2.11 | 0.82 | 0.11 |
|  | Ommastrephidae | |  |  |  |  |
|  |  | *Nototodarus* sp. | 0.47 | 0.09 | 0.27 | <0.01 |
|  | Cranchiidae | |  |  |  |  |
|  |  | Cranchiidae unidentified | 0.47 | 1.19 | 0.27 | 0.02 |
|  | Teuthoidea unidentified | | 2.34 | 6.14 | 1.36 | 0.46 |
| **Other** | | |  |  |  |  |
|  | Crustacea unidentified | | 0.47 | <0.01 | 0.54 | 0.01 |
|  | Cephalopoda unidentified | | 1.87 | 0.47 | 1.09 | 0.08 |
|  | Eumalacostraca unidentified | | 2.34 | <0.01 | 1.36 | 0.08 |
|  | Scales | | 14.95 | 0.03 | 8.72 | 3.45 |
|  | Otoliths | | 6.07 | 0.01 | 4.36 | 0.70 |
|  | Fishes unidentified | | 41.59 | 37.56 | 31.88 | 76.22 |
|  | Unidentifiable | | 1.87 | 0.02 | 0.82 | 0.04 |
|  |  |  |  |  |  |  |

Bold text lines show the point estimates, and 95% confidence intervals estimated by Bootstrap resampling, of the percentage frequency of occurrence (%F), percentage weight (%W), percentage number (%N), and percentage Index of Relative Importance (%IRI), for prey grouped at the taxonomic levels used in the multivariate analyses (number of stomachs = 133). Under each prey group, the normal text lines show the point estimates of the dietary statistics when calculated for all prey types (i.e. at full resolution). Abiotic material and prey types that could not be allocated to one of the prey groups (so excluded from multivariate analyses) are listed at the bottom of the table.
