# Supplementary material for: Trophic Interactions and Distribution of Some Squaliforme Sharks, Including New Diet Descriptions for Deania calcea and Squalus acanthias
Source: PLoS One. 2013 Mar 25;8(3):e59938. doi: 10.1371/journal.pone.0059938 (PMC3607562; doi:10.1371/journal.pone.0059938)
Supplement: Table S2 — Stomach contents composition for Squalus acanthias from the Chatham Rise 2005, 2006 and 2007 combined. (DOCX) [file pone.0059938.s002.docx]

**Table S2:** Stomach contents composition for *Squalus acanthias* from the Chatham Rise 2005, 2006 and 2007 combined.

|  |  |  | %F | %W | %N | %IRI |
| --- | --- | --- | --- | --- | --- | --- |
|  |  |  |  |  |  |  |
| **Cnidaria** | | | **0.3 (0–1.5)** | **<0.1 (0–0.03)** | **0.1 (0–0.2)** | **<0.1 (0–0.01)** |
|  | Scyphozoa unidentified | | 0.21 | <0.01 | 0.04 | <0.01 |
| **Salpida** | | | **19.4 (12.1–27.3)** | **0.9 (0.2–2.1)** | **48.3 (19.4–74.2)** | **36.7 (9.0–61.3)** |
|  |  | Salpida unidentified | 11.73 | 0.62 | 41.15 | 16.96 |
| **Mollusca** | | |  |  |  |  |
| **Sepiolida** | | | **0.7 (0–2.4)** | **<0.1 (0–0.1)** | **0.2 (0–0.7)** | **<0.1 (0–0.1)** |
|  | Sepiolidae | |  |  |  |  |
|  |  | *Stoloteuthis maoria* | 0.41 | 0.01 | 0.13 | 0.01 |
| **Teuthoidea** | | | **16 (9.7–24.0)** | **25.8 (11.3–43.9)** | **2.6 (1.2–6.6)** | **17.5 (5.4–37.1)** |
|  | Ommastrephidae | |  |  |  |  |
|  |  | *Nototodarus sloani* | 0.21 | 0.22 | 0.04 | <0.01 |
|  |  | *N.* sp. | 3.70 | 12.61 | 0.87 | 1.73 |
|  | Onychoteuthidae | |  |  |  |  |
|  |  | *Onykia ingens* | 0.41 | 0.03 | 0.09 | <0.01 |
|  | Cranchiidae | |  |  |  |  |
|  |  | Cranchiidae unidentified | 0.21 | 0.01 | 0.04 | <0.01 |
|  | Teuthoidea unidentified | | 5.35 | 6.05 | 1.17 | 1.34 |
| **Octopoda** | | | **3.7 (1.4–6.9)** | **10.5 (0.3–23.2)** | **0.6 (0.2–1.7)** | **1.6 (0.1–5.3)** |
|  | Octopoda unidentified | | 2.26 | 7.71 | 0.52 | 0.64 |
| **Polychaeta** | | | **2.4 (0.4–5.2)** | **0.3 (0.01–1.0)** | **0.4 (0.1–1.2)** | **0.1 (0–0.4)** |
|  | Eunicidae | |  |  |  |  |
|  |  | *Eunice* spp. | 0.62 | 0.20 | 0.13 | 0.01 |
|  | Onuphidae | | 0.21 | 0.01 | 0.04 | <0.01 |
|  | Polychaeta unidentified | | 0.62 | 0.03 | 0.17 | <0.01 |
| **Pycnogonida** | | | **0.3 (0–1.5)** | **<0.1** | **0.1 (0–0.5)** | **<0.1 (0–0.02)** |
|  |  | Pycnogonida unidentified | 0.21 | <0.01 | 0.09 | <0.01 |
| **Crustacea** | | |  |  |  |  |
| **Ostracoda** | | | **1.0 (0–2.8)** | **<0.1 (0–7.8)** | **0.3 (0–1.0)** | **<0.1 (0–0.1)** |
|  |  | Ostracod unidentified | 0.62 | <0.01 | 0.22 | <0.01 |
| **Copepoda** | | | **10.5 (2.4–20.2)** | **<0.1 (0–0.1)** | **2.8 (0.6–6.9)** | **1.1 (0.05–5.0)** |
|  | Calanoida unidentified | | 6.38 | <0.01 | 2.34 | 0.52 |
| **Stomatopoda** | | | **0.3 (0–1.4)** | **<0.1 (0–0.03)** | **0.1 (0–0.3)** | **<0.1 (0–0.01)** |
|  |  | Stomatopoda unidentified | 0.21 | <0.01 | 0.04 | <0.01 |
| **Euphausiacea** | | | **17 (7.7–28.7)** | **0.1 (0–0.2)** | **33.4 (5.1–61.6)** | **21.9 (1.7–55.1)** |
|  | Euphausiidae | |  |  |  |  |
|  |  | *Nyctiphanes australis* | 5.14 | 0.05 | 17.14 | 3.06 |
|  |  | *Thysanoessa gregaria* | 0.21 | <0.01 | 0.04 | <0.01 |
|  | Euphausiacea unidentified | | 7.00 | 0.01 | 11.24 | 2.73 |
| **Natant Decapoda** | | | **4.1 (1.0–7.8)** | **0.1 (0.1–18.4)** | **0.8 (0.2–2.2)** | **0.1 (0.01–0.7)** |
|  | Crangonidae | | 0.21 | <0.01 | 0.04 | <0.01 |
|  | Pandalidae | |  |  |  |  |
|  |  | *Notopandalus magnoculus* | 0.41 | 0.01 | 0.09 | <0.01 |
|  | Oplophoridae | |  |  |  |  |
|  |  | *Oplophorus novaezeelandiae* | 0.21 | 0.01 | 0.04 | <0.01 |
|  | Natant decapoda unidentified | | 1.65 | 0.03 | 0.48 | 0.03 |
| **Reptant Decapoda** | | | **6.1 (2.5–10.6)** | **2.2 (0.5–5.2)** | **1.0 (0.4–2.6)** | **0.8 (0.1–2.5)** |
| **Astacidea** | | |  |  |  |  |
|  | Nephropidae | |  |  |  |  |
|  |  | *Metanephrops challengeri* | 1.03 | 0.93 | 0.22 | 0.04 |
| **Palinura** | | |  |  |  |  |
|  | Palinuridae | |  |  |  |  |
|  |  | Phyllosoma unidentified | 0.21 | <0.01 | 0.04 | <0.01 |
| Crab unidentified | | | 0.82 | 0.11 | 0.17 | 0.01 |
| **Anomura** | | |  |  |  |  |
|  | Galatheidae | |  |  |  |  |
|  |  | *Munida gracilis* | 0.41 | 0.04 | 0.17 | <0.01 |
|  |  | *M.* spp. | 0.21 | 0.01 | 0.04 | <0.01 |
| **Brachyura** | | |  |  |  |  |
|  | Atelecyclidae | |  |  |  |  |
|  |  | *Trichopeltarion fantasticum* | 0.41 | 0.23 | 0.09 | <0.01 |
|  | Goneplacidae | |  |  |  |  |
|  |  | *Neommatocarcinus huttoni* | 0.21 | 0.01 | 0.04 | <0.01 |
|  | Majidae | |  |  |  |  |
|  |  | *Leptomithrax* spp. | 0.41 | 0.27 | 0.09 | 0.01 |
| **Amphipoda** | | | **9.9 (4.9–15.5)** | **<0.1 (0–0.02)** | **3.3 (1.3–6.9)** | **1.3 (0.3–3.2)** |
| Gammaridea | | |  |  |  |  |
|  | Gammaridea unidentified | | 4.94 | 0.01 | 2.43 | 0.42 |
| Hyperiidea | | |  |  |  |  |
|  | Cyllopodidae | |  |  |  |  |
|  |  | *Cyllopus magellanicus* | 0.62 | <0.01 | 0.17 | <0.01 |
|  | Hyperiidae | |  |  |  |  |
|  |  | *Themisto gaudichaudii* | 0.41 | <0.01 | 0.22 | <0.01 |
| **Isopoda** | | | **0.3 (0–1.2)** | **<0.1 (0–0.07)** | **0.1 (0–0.2)** | **<0.1 (0–0.01)** |
|  | Isopoda unidentified | | 0.21 | 0.01 | 0.04 | <0.01 |
| **Echinodermata** | | | **0.7 (0–2.3)** | **<0.1 (0–0.01)** | **0.1 (0–0.4)** | **<0.1 (0–0.03)** |
|  | Ophiuroidea | | 0.21 | <0.01 | 0.04 | <0.01 |
|  | Echinoidea | | 0.21 | <0.01 | 0.04 | <0.01 |
| **Chondrichthyes** | | | **0.3 (0–1.7)** | **0.2 (0–1.1)** | **<0.1 (0–0.3)** | **<0.1 (0–0.1)** |
|  | Chimaeridae | |  |  |  |  |
|  |  | *Hydrolagus novaezelandiae* | 0.21 | 0.16 | 0.04 | <0.01 |
| **Osteichthyes** | | |  |  |  |  |
|  | **Argentinidae** | | **2.4 (0.6–4.9)** | **2.8 (0.5–6.6)** | **0.5 (0.1–1.5)** | **0.3 (0–1.3)** |
|  |  | *Argentina elongata* | 1.44 | 2.07 | 0.43 | 0.12 |
|  | **Sternoptychidae** | | **0.7 (0–2.3)** | **<0.1 (0–0.03)** | **0.1 (0–0.5)** | **<0.1 (0–0.04)** |
|  |  | *Maurolicus australis* | 0.41 | 0.01 | 0.09 | <0.01 |
|  | **Myctophidae** | | **4.4 (1.9–7.8)** | **0.2 (0.02–0.06)** | **0.8 (0.2–2.1)** | **0.2 (0.02–0.7)** |
|  |  | *Lampanyctodes hectoris* | 0.41 | 0.01 | 0.09 | <0.01 |
|  |  | *Lampanyctus* spp. | 0.21 | 0.04 | 0.04 | <0.01 |
|  |  | *Lampichthys procerus* | 0.21 | 0.02 | 0.04 | <0.01 |
|  |  | *Symbolophorus* *boops* | 0.41 | 0.08 | 0.09 | <0.01 |
|  |  | Myctophidae unidentified | 1.65 | 0.01 | 0.39 | 0.02 |
|  | **Moridae** | | **1.0 (0–2.8)** | **3.4 (0–11.5)** | **0.2 (0–0.6)** | **0.1 (0–1.2)** |
|  |  | *Antimora rostrata* | 0.21 | 1.73 | 0.04 | 0.01 |
|  |  | *Austrophycis marginata* | 0.21 | 0.01 | 0.04 | <0.01 |
|  |  | *Pseudophycis bachus* | 0.21 | 0.77 | 0.04 | 0.01 |
|  | **Merlucciidae** | | **4.4 (1.7–8.0)** | **20.4 (4.7–37.4)** | **0.8 (0.2–2.2)** | **3.6 (0.4–10.9)** |
|  |  | *Macruronus novaezelandiae* | 2.67 | 14.97 | 0.65 | 1.45 |
|  | **Macrouridae** | | **8.2 (4.2–12.9)** | **7.8 (1.7–18.3)** | **1.3 (0.5–3.2)** | **2.9 (0.4–8.5)** |
|  |  | *Coelorinchus aspercephalus* | 0.21 | 0.56 | 0.04 | <0.01 |
|  |  | *C. oliverianus* | 1.23 | 0.29 | 0.26 | 0.02 |
|  |  | *Lepidorhynchus denticulatus* | 1.44 | 3.78 | 0.30 | 0.20 |
|  |  | *Coryphaenoides* spp. | 0.21 | 0.10 | 0.04 | <0.01 |
|  |  | Macrouridae unidentified | 2.06 | 0.98 | 0.43 | 0.10 |
|  | **Ophidiidae** | | **0.3 (0–1.5)** | **0.1 (0–0.4)** | **0.1 (0–0.3)** | **<0.1 (0–0.04)** |
|  |  | *Genypterus blacodes* | 0.21 | 0.06 | 0.04 | <0.01 |
|  | **Zeidae** | | **0.3 (0–1.6)** | **<0.1 (0–0.1)** | **0.1 (0–0.3)** | **<0.1 (0–0.02)** |
|  |  | *Capromimus abbreviatus* | 0.21 | 0.01 | 0.04 | <0.01 |
|  | **Oreosomatidae** | | **0.3 (0–1.4)** | **0.2 (0–0.8)** | **0.1 (0–0.3)** | **<0.1 (0–0.1)** |
|  |  | Oreosomatidae unidentified | 0.21 | 0.13 | 0.04 | <0.01 |
|  | **Macrorhamphosidae** | | **0.7 (0–2.2)** | **0.1 (0–0.5)** | **0.1 (0–0.5)** | **<0.1 (0–0.1)** |
|  |  | *Centriscops* spp. | 0.41 | 0.09 | 0.09 | <0.01 |
|  | **Scorpaenidae** | | **0.3 (0–1.5)** | **0.4 (0–2.0)** | **0.1 (0–0.3)** | **<0.1 (0–0.1)** |
|  |  | *Helicolenus* spp. | 0.21 | 0.31 | 0.04 | <0.01 |
|  | **Hoplichthyidae** | | **0.7 (0–2.2)** | **<0.1 (0–0.01)** | **0.1 (0–0.4)** | **<0.1 (0–0.03)** |
|  |  | *Hoplichthys haswelli* | 0.41 | <0.01 | 0.09 | <0.01 |
|  | **Serranidae** | | **0.3 (0–1.4)** | **0.8 (0–3.4)** | **0.1 (0–0.3)** | **<0.1 (0–0.2)** |
|  |  | *Lepidoperca aurantia* | 0.21 | 0.58 | 0.04 | <0.01 |
|  | **Emmelichthyidae** | | **0.3 (0–1.6)** | **0.6 (0–2.8)** | **0.1 (0–0.3)** | **<0.1 (0–0.2)** |
|  |  | *Emmelichthys nitidus* | 0.21 | 0.41 | 0.04 | <0.01 |
|  | **Discarded fish** | | **12.2 (6.2–18.9)** | **23.1 (10.9–39.0)** | **1.9 (0.9–4.7)** | **11.8 (3.0–25.3)** |
|  |  | *Macruronus novaezelandiae* | 0.41 | 1.22 | 0.09 | 0.02 |
|  |  | *Trachurus* spp. | 7.00 | 15.7 | 1.56 | 4.18 |
| **Other** | | |  |  |  |  |
|  | Algae unidentified | | 0.21 | 0.04 | 0.04 | <0.01 |
|  | Cephalopoda unidentified | | 1.23 | 2.88 | 0.26 | 0.13 |
|  | Crustacea unidentified | | 1.03 | 0.03 | 0.22 | 0.01 |
|  | Eumalacostraca unidentified | | 0.41 | <0.01 | 0.13 | <0.01 |
|  | Fishes unidentified | | 51.65 | 23.65 | 13.32 | 66.09 |
|  | Sand | | 1.23 | 0.01 | 0.26 | 0.01 |
|  | Unidentifiable | | 3.09 | 0.03 | 0.61 | 0.07 |

Bold text lines show the point estimates, and 95% confidence intervals estimated by Bootstrap resampling, of the percentage frequency of occurrence (%F), percentage weight (%W), percentage number (%N), and percentage Index of Relative Importance (%IRI), for prey grouped at the taxonomic levels used in the multivariate analyses (number of stomachs = 295). Under each prey group, the normal text lines show the point estimates of the dietary statistics when calculated for all prey types (i.e. at full resolution). Abiotic material and prey types that could not be allocated to one of the prey groups (so excluded from multivariate analyses) are listed at the bottom of the table.
